# Femtosecond Laser Inscribed Excessively Tilted Fiber Grating for Humidity Sensing

**DOI:** 10.3390/s24020342

**Published:** 2024-01-06

**Authors:** Liqing Jing, Bonan Liu, Dejun Liu, Dan Liu, Famei Wang, Chunying Guan, Yiping Wang, Changrui Liao

**Affiliations:** 1 Key Laboratory of In-Fiber Integrated Optics of Ministry of Education, College of Physics and Optoelectronic Engineering, Harbin Engineering University, Harbin 150001, China; jlq@szu.edu.cn (L.J.); cyguan@163.com (C.G.); 2 Key Laboratory of Optoelectronic Devices and Systems of Ministry of Education and Guangdong Province, College of Physics and Optoelectronic Engineering, Shenzhen University, Shenzhen 518061, Chinacliao@szu.edu.cn (C.L.); 3 Shenzhen Key Laboratory of Photonic Devices and Sensing Systems for Internet of Things, Guangdong and Hong Kong Joint Research Centre for Optical Fiber Sensors, Shenzhen University, Shenzhen 518060, China

**Keywords:** excessively tilted fiber grating sensing, agarose sensors, humidity sensing, femtosecond laser processing

## Abstract

We propose a humidity sensor using an excessively tilted fiber grating (Ex-TFG) coated with agarose fabricated using femtosecond laser processing. The processed grating showcases remarkable differentiation between TE and TM modes, achieving an exceptionally narrow bandwidth of approximately 1.5 nm and an impressive modulation depth of up to 15 dB for both modes. We exposed the agarose-coated TFG sensor to various relative humidity levels and monitored the resonance wavelength to test its humidity sensing capability. Our findings demonstrated that the sensor exhibited a rapid response time (2–4 s) and showed a high response sensitivity (18.5 pm/%RH) between the humidity changes and the resonant wavelength shifts. The high sensitivity, linearity, repeatability, low hysteresis, and excellent long-term stability of the TFG humidity sensor, as demonstrated in our experimental results, make it an attractive option for environmental monitoring or biomedical diagnosis.

## 1. Introduction

Humidity sensing plays a vital role in monitoring human health, precision machining, and environmental measurements, leading to the development of various humidity detection devices [1]. Optical fiber humidity sensors (OFHSs) have emerged as the preferred choice due to their unique advantages such as long lifespan, non-corrosiveness, light weight, resistance to electromagnetic interference, and remote sensing. A number of fiber structures have been proposed for humidity sensing based on different working principles, such as gratings [2,3], interferometers [4], Fabry–Pérot cavities [5] and resonators [6].

Among the different types of fiber optic humidity sensors, grating-based devices have shown great promise. Gratings are periodic structures fabricated on the fiber core, enabling the interaction of light with the surrounding environment. In recent years, fiber optic grating technology has evolved to include several grating types, including fiber Bragg grating (FBG) [7,8,9], long-period fiber grating (LPFG) [10] and tilted fiber grating (TFG) [11,12]. Compared with the above-mentioned optical fiber grating, the excessively tilted fiber grating (Ex-TFG) shows a series of orthogonal polarization dual peaks in the transmission spectrum and exhibits a high surrounding refractive index (SRI) sensitivity with low temperature crosstalk. This unique feature positions Ex-TFGs as strong contenders for refractive index-related sensing applications. Together with these advancements in grating-based fiber optic sensors, Ex-TFGs offer a promising avenue for both humidity and refractive index sensing applications.

Humidity sensing can be achieved with a tilted FBG (TFBG) by depositing a moisture material on its surface. Variations in environmental humidity introduce the expansion of the moisture material and changes in RI, eventually resulting in changes of the modes coupling in TFBG and a shift in the spectra [13]. Polymers such as polyimide and polyvinyl alcohol are sensitive to humidity but have a slow response and significant hysteresis [14,15,16]. Agarose is a popular material due to its high water absorption capacity, low toxicity, biocompatibility [17,18] and stability in aqueous environments. Its hydrogel layer can absorb water, changing the RI of the cladding and resulting in a shift in the TFG’s resonant wavelength. Agarose is cost effective, obtained from seaweed, and more sensitive than PVA or polyimide. Its biocompatibility also makes it suitable for biomedical applications such as monitoring humidity in biological samples.

In this work, a relative humidity (RH) sensor with high performance is proposed. The sensor is developed based on an Ex-TFG fabricated using femtosecond laser direct inscription and agarose coating. The Ex-TFG used has a large tilt angle of 81° that breaks the fiber’s directional symmetry, exciting a set of polarization-related cladding modes. By tracking the resonant wavelength of specific cladding modes, the agarose-deposited TFG shows a humidity sensitivity of 18.5 pm/%RH, a quick response (2–4 s), good linearity and low hysteresis. The sensor exhibits great potential to be applied in various fields such as pharmaceutical processes, human health, and environmental monitoring.

## 2. TFG Fabrication and Sensing Principle

**Figure 1:**
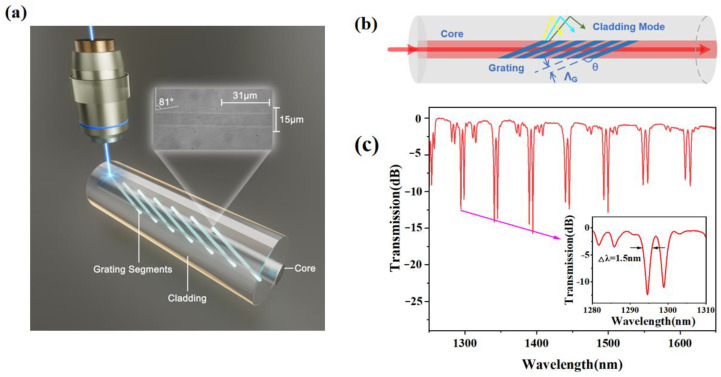
(**a**) Schematic diagram of the fabrication. Inset: microscope image of the obtained TFG. (**b**) Schematic diagram of Ex-TFG coupling principle. (**c**) The transmission spectrum of a TFG. Inset: enlarged view of the dual resonance.

TFG performance is closely related to the tilt angle, requiring precise control during manufacturing to achieve satisfactory results. Femtosecond laser direct writing technology [19,20] offers several advantages, such as flexibility in the design of TFG structures and the ability to fabricate TFGs in various fibers. The high precision of femtosecond laser processing allows for narrow bandwidths and reproducibility, making it reliable for large-scale production. To ensure precise and flexible control of the fabrication, femtosecond laser direct inscription is used herein with the tilt angle and the grating period selected as 81° and 31 um [21,22,23], respectively. Figure 1a illustrates the femtosecond laser fabrication setup. The grating is inscribed in a single-mode fiber (YOFC, SMF-28) using a line-by-line carving method [24,25]. To achieve precise fabrication, we utilized an air-bearing translation stage (Aerotech) to mount the fiber and control its motion during the process. The femtosecond laser operating at 515 nm with a pulse duration of 290 fs and an average power of 1.14 mW generated optical pulses at a repetition rate of 100 kHz with a pulse energy of 1.14 × 10^−8^ J (11.4 nJ). The optical pulse was focused onto the fiber using an oil-immersion 100× objective lens with a numerical aperture of 1.32, and the fiber coating was pre-removed. The TFGs were inscribed line by line along a straight line passing through the fiber core. Figure 1a inset shows the optical microscope image of the tilted stripes in the TFG core inspected using a high-resolution microscope.

The light coupling mechanism of the Ex-TFG is demonstrated in Figure 1b. The Ex-TFG has a similar grating period to that of LPG, but its grating plane is tilted at an angle relative to the fiber axis, breaking the circular symmetry of the fiber. The fiber cross-section’s asymmetry causes the Ex-TFG to display a noticeable dual-peak feature with multiple notches. Each coupling cladding mode has two degenerate modes, the fast and slow axes. A polarization controller can selectively excite the TM mode, the TE mode, or both degenerate modes [21,26,27].

The phase matching condition can be expressed as follows [28]:

$$\lambda =\left(n_{eff}^{co}-n_{i,eff}^{cl,m}\right)\frac{\Lambda_{G}}{\cos\theta }$$

where i represents the resonance of the TE or TM modes, which are the orthogonal modes with a period $\Lambda_{G}$ perpendicular to the grating plane, θ is the tilt angle, and $n_{eff}^{co}$ and $n_{i,eff}^{cl,m}$ represent the effective refractive indices (ERI) of the core mode and the mth cladding mode, respectively. The RI of the surrounding medium can affect the ERI of the cladding mode, thereby affecting the resonant wavelength. This forms the fundamental principle of environmental RI sensing.

A typical obtained grating spectrum is shown in Figure 1c. The processed grating can clearly distinguish between the TE and TM modes with a narrow bandwidth of approximately 1.5 nm for each. Moreover, the grating modulation depth reaches up to 15 dB. These improvements greatly enhance the efficiency of fabricating large-angle tilted gratings using femtosecond laser processing and increase their sensitivity to environmental changes.

**Figure 2:**
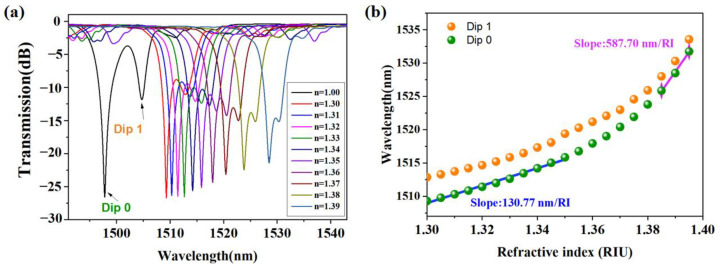
(**a**) Spectral evolution of the Ex-TFG with the ambient RIs. (**b**) Wavelength shifts of Dip 0 and Dip 1 versus ambient RIs.

The RI response of the Ex-TFGs was tested with RI matching liquid ranging from 1.30 to 1.39. Figure 2a shows the spectral evolution of an Ex-TFG. The left dip is known as the TM mode while the right one is the TE mode. From the figure, it can be observed that as the RI of the liquid increases, the peak of the signal gradually decreases which indicates that the cladding mode extends into the radiation mode.

The corresponding wavelength shifts for both the TE and TM modes were extracted and plotted in Figure 2b. As the surrounding RI varies, the wavelength is observed to shift towards longer wavelengths, spanning slightly over 20 nm throughout the entire measurement. As depicted, the grating exhibits a sensitive RI response within the range of 1.30–1.39, with response sensitivities of approximately 130.77 nm/RI and 587.70 nm/RI at RI ranges of 1.30–1.35 and 1.385–1.395, respectively. These response sensitivities are beneficial for RI sensing applications. This high response sensitivity is attributed to the change in the effective RI of the fiber core mode and the cladding mode, which is caused by changes in the surrounding RI. The sensitivity of the Ex-TFG to the RI of the surrounding medium can be optimized by adjusting the grating period, the tilt angle, and the coating thickness. The use of Ex-TFG for RI sensing has a wide range of potential applications, including environmental monitoring, biological sensing, and chemical sensing.

## 3. Surface Modification and Characterization

In order to achieve a sensitive and fast response to RH levels, a layer of coating containing hydrophilic groups needs to be deposited on the surface of the Ex-TFG. Agarose gel is a suitable material for humidity sensing due to its desirable sensitivity to changes in moisture levels [29,30]. This is because it can easily absorb and release water molecules, allowing it to quickly reach equilibrium with the surrounding atmospheric moisture. Agarose is a polysaccharide that forms a gel when dissolved in water, making it an ideal material for creating a stable hydrogel layer on the Ex-TFG surface. 

**Figure 3:**
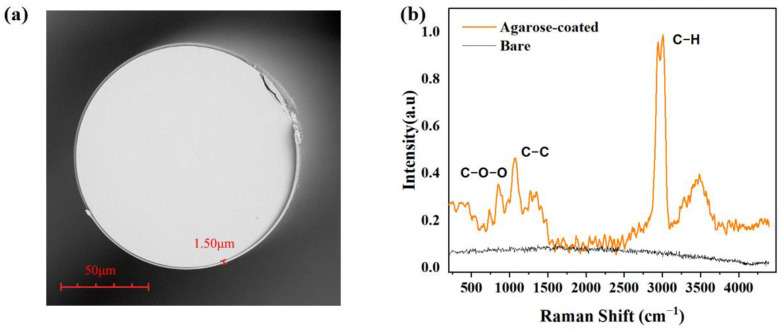
(**a**) SEM image of the cross section of a coated Ex-TFG. (**b**) Raman spectra of bare and agarose-coated TFG.

AGAROSEG-10 (BIOWEST) was used for surface modification. Furthermore, 1 mg of agarose was dissolved in 100 mL of deionized water under stirring at 100 °C until an agarose solution was formed. Then, the ex-TFG was coated with the agarose solution using the gel droplet coating method, and a uniform agarose film was obtained as shown in Figure 3a. To characterize the coating, Raman spectra of the bare Ex-TFG and the coated one were measured. As shown in Figure 3b, peaks are observed around 857 cm^−1^, 1074 cm^−1^, and 2944 cm^−1^ for a sample coated with evenly and effectively deposited agarose. The 857 cm^−1^ peak is attributed to the vibration of the C–O–C bond in agarose molecules, known as the “chain planar bending mode”; the 1074 cm^−1^ peak is attributed to the vibration of the C–C bond in agarose molecules, known as the “β-chain twisting mode”; and the 2944 cm^−1^ peak is attributed to the vibration of the C–H bond in agarose molecules, known as the “symmetric stretching mode of methylene groups”. It can be concluded that agarose was well deposited on the surface of the Ex-TFG.

**Figure 4:**
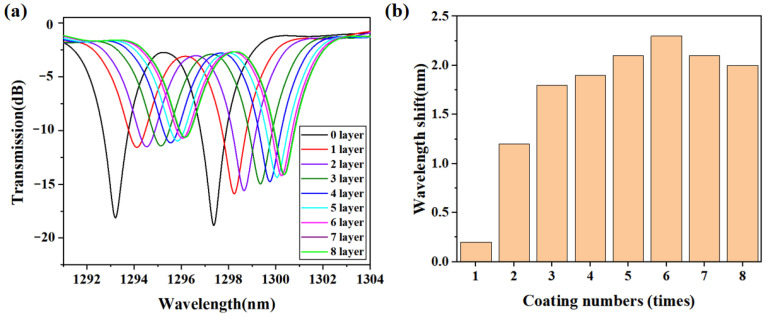
(**a**) Spectral evolution of the Ex-TFG with different layers of agarose deposition. (**b**) The corresponding wavelengths shift as a function of the deposition layers.

After each deposition of the agarose, the tilted grating was fixed and tested for humidity response. Figure 4a illustrates the spectral evolution of the Ex-TFG (tilted fiber grating) under the same humidity conditions with varying thicknesses of agarose deposition. The laboratory environment had a humidity of about 50%, while the humidity of exhaled breath from a human mouth was about 95%. We compared the spectral wavelength shift of the gratings with different thicknesses of deposited agarose after exposure to both the laboratory environment and human breath. The experimental results showed that the humidity response of the sensor increased with increasing thickness of the agarose film up to a certain point, after which it leveled off. The increase in humidity response with increasing film thickness was attributed to the increased surface area for water absorption and the corresponding increase in RI change of the sensor. However, beyond a certain thickness, the response of the sensor became saturated due to the limited diffusion of water molecules through the thick film. From Figure 4b, it is evident that the maximum wavelength shift of the sensor occurs at around six layers of agarose deposition. Hence, for the humidity sensing experiment herein, the sensor was coated with six layers of agarose. This strategic control over agarose thickness not only enhances our understanding of the sensor’s behavior, but also paves the way for improved sensor design and performance in various applications. 

## 4. Results and Discussion

### 4.1. Sensitivity of the RH Sensor

**Figure 5:**
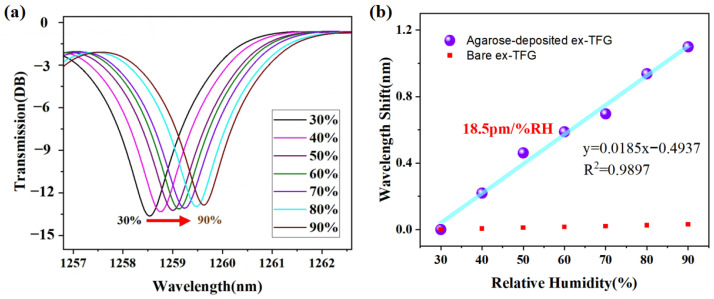
(**a**) Spectral evolution of the agarose-deposited Ex-TFG with varied RH levels; (**b**) resonant wavelength shifts versus the RH level.

To detect the relative humidity (RH) response, the fabricated sensor was fixed and sealed in a temperature and humidity control box (DHT, DHTHM-27). The light from a broadband light source (BBS) was transmitted through the sensor, and the transmitted spectrum was monitored using an optical spectrum analyzer (OSA). The humidity chamber ranged from 20% to 100% RH. During the measurements, the RH was increased from 30% to 90% and then gradually decreased back to its initial state. To avoid the influence of temperature on RH measurements, the temperature in the humidity chamber was maintained at ~25 °C during the experimental process. Figure 5a shows the spectral evolution of the sensor at different RH levels. As the RH increases, the agarose film absorbs more water vapor, causing an increase in film RI and a red shift in the resonance wavelength. As shown in Figure 5b, within the 30% to 90% RH range, the extracted resonance wavelength shows a linear increase with increasing RH, and the response sensitivity obtained is 18.5 pm/%RH with a linearity of 98.97%. Compared to the bare Ex-TFG, the RH sensitivity of the sensor is significantly higher and remains relatively constant as RH varies.

The sensing enhancement of TFG with agarose deposition can be explained by the permeation mechanism of water molecules into the agarose layer. Agarose has a highly porous and hydrophilic structure with a large number of hydroxyl (-OH) groups present on the surface of the agarose molecules. These hydroxyl groups can form hydrogen bonds with water molecules, allowing for the rapid diffusion of water molecules into and out of the agarose film. The porous structure of agarose also allows for an increased surface area for water absorption, which can further enhance the sensitivity of the TFG sensor to changes in humidity. When moisture is present in the surrounding environment, water molecules can quickly and easily diffuse into the agarose film, causing material expansion and a change in the RI, which can be detected and measured by monitoring changes in transmission spectrum.

### 4.2. The Response Time and Stability

**Figure 6:**
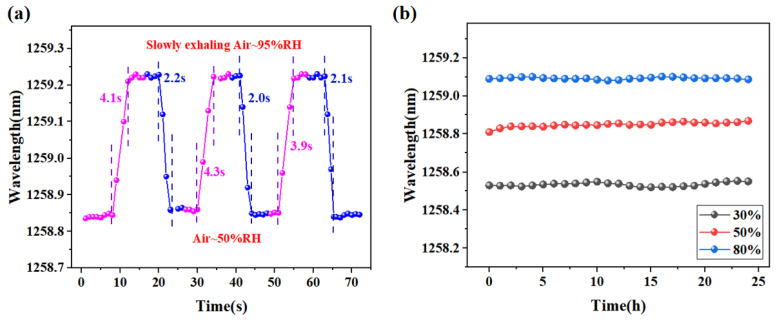
(**a**) Response time test of the RH sensor. (**b**) Stability test of the proposed sensor.

To investigate the time response of the sensor, a step-change experiment was conducted. The RH surrounding the sensor was rapidly changed from a low level to a high level (from 50% to 95%), and the response of the sensor was recorded over time until it reached a steady state. The same experiment was also conducted in the opposite direction, from a high humidity level to a low humidity level (from 95% to 50%). The time response of the sensor was then analyzed based on the recorded data. Furthermore, it was discovered that the absorption and desorption of moisture is a reversible process that varies with the RH. It was conducted in the same way as the experiment investigating the relationship between the humidity response and the thickness of the agarose film. The low humidity condition was set to the humidity level of the laboratory environment (about 50%), and the high humidity condition was set to the humidity level of human mouth exhalation (about 95%). The experimental results are shown in Figure 6a. As can be seen from the graph, it takes about 4 s to reach stability when the humidity increases from 50% to 95%, while it takes about 2 s to reach stability when the humidity decreases from 95% to 50%. This indicates that the rate of release of water molecules from the agarose is greater than the rate of absorption of water molecules. Furthermore, it was discovered that the absorption and desorption of moisture is a reversible process that varies with the RH.

In addition to studying the humidity testing stability of the humidity sensor, we also assessed its overall stability under varying humidity conditions. To evaluate its performance, the sensor was subjected to a series of humidity control boxes set to different humidity levels, specifically 30%, 50%, and 80%. Throughout the 24 h experimental period, the sensor’s response was continuously monitored, and the corresponding spectra were recorded every hour. The results, as depicted in Figure 6b, reveal that the sensor exhibited remarkable stability during the entire testing duration. Notably, the sensor’s response remained consistent under different humidity conditions, indicating its ability to maintain accurate and reliable readings over time. This consistent performance signifies the sensor’s exceptional stability, bolstering confidence in its long-term effectiveness and suitability for practical applications.

### 4.3. Discussion

The humidity sensor presented in this study, based on the Ex-TFG and coated with an agarose film, exhibits distinct advantages, as highlighted in Table 1.

In comparison to other reported sensors, those relying on Pyralin, PVA, PMMA, or polyimide, either based on or coated with FBG/TFBG, demonstrate low sensitivity [15,31,32]. Moreover, they struggle to precisely monitor environmental humidity. Sensors utilizing GO-coated etched surfaces, polyimide, and polymers exhibit response times exceeding 10 min [33,34,35]. While AGAROSEG-coated No-Core sensors boast high sensitivity, their testing range is limited [36].

In contrast, our proposed relative humidity (RH) sensor demonstrates superior characteristics, including high sensitivity and a rapid response time, within a broad dynamic RH range. The outstanding performance of our developed sensor positions it as a promising candidate for various applications, particularly in the field of medical science. Specifically, the sensor shows potential for precision medicine applications, particularly in the realm of respiratory diseases. The reliable and accurate monitoring of humidity levels can contribute significantly to enhancing the precision and efficacy of medical interventions for respiratory conditions.

## 5. Conclusions

In this study, a highly sensitive humidity sensor based on an Ex-TFG was proposed and experimentally verified. The unique features of the Ex-TFG fabrication process using femtosecond laser processing with optimized parameters allow for high spectral resolution. By depositing an appropriate thickness of agarose on the Ex-TFG surface, a strong interaction between the cladding mode resonance and the layer can be achieved. The transmission spectrum of the Ex-TFG shows a linear red shift with increasing relative humidity due to moisture absorption changing the effective RI of the cladding mode. Our results demonstrate a quick response (2–4 s) and a high response sensitivity (18.5 pm/%RH) between the humidity level and the shift in the resonant wavelength. The experimental findings also showcase high repeatability and excellent long-term stability during continuous measurements across a wide dynamic range of relative humidity. The TFG humidity sensor’s outstanding attributes, as demonstrated in our experiments, make it an appealing choice for environmental monitoring and biomedical diagnosis.

## Figures and Tables

**Table 1:** Comparisons of the Optical Fiber RH Sensors.

Sensor Type	RH Range (% RH)	Sensitivity (pm/%RH)	Response	Recovery	Ref
Pyralin-coated-FBG	15−95	1.28	5 s	20 s	[31]
PVA-coated hybrid fiber grating	30–95	Nonlinear, ~0.737 nW/%RH	~2 s	NA	[32]
GO-coated etched TFBG	20–80	10	12.25 min	21.75 min	[33]
Polyimide-coated FBG	12–97	13.6	22 min	NA	[34]
Polyimide-coated FBG	20−90	1.71	>33 s	NA	[15]
Polymer FBG	50–95	35	30 min	NA	[35]
AGAROSEG-coated No-Core	30–75	149	4.8 s	7.1 s	[36]
AGAROSEG-coated TFBG	30–95	18.5	4 s	2 s	Our work

## Data Availability

Data are available from the corresponding author and can be provided upon appropriate request.
